# Depression and anxiety among medical students in St. Petersburg, Russia: comparison of data from 2024 and 2020

**DOI:** 10.1192/j.eurpsy.2026.10793

**Published:** 2026-07-31

**Authors:** E. Chumakov, P. Makarova, I. Pchelin, N. Petrova

**Affiliations:** 1 Department of Psychiatry and Addiction; 2 Department of Faculty Therapy, St. Petersburg State University, St. Petersburg, Russian Federation

## Abstract

**Introduction:**

The mental health of medical students in Russia remains insufficiently studied, with a lack of multicenter and longitudinal research. Our early research during the COVID-19 pandemic showed high levels of anxiety and depression symptoms among medical students in St. Petersburg, Russia, with no gender differences.

**Objectives:**

The aim of the study was to evaluate the dynamics of the manifestations and severity of depression and anxiety symptoms in medical students studying at the same institute.

**Methods:**

An anonymous structured online survey was conducted among students of a medical institute in St. Petersburg, Russia, in June-July 2024. The Hospital Anxiety and Depression Scale (HADS) was used for screening anxiety and depression symptoms. The final sample consisted of 141 students (77.3% females). The results were compared with the data of a survey conducted at the same institute in 2020 (Chumakov et al. Int J Soc Psychiatry 2022; 68(6) 1270-1276).

**Results:**

In 2024, 52 students (36.9%) reported that they had experienced the negative impact of the COVID-19 pandemic on their psychological well-being, and 30 students (21.3%) reported that they felt that their mental state had not improved after the end of the pandemic. Clinical or subclinical symptoms of anxiety according to HADS were reported by 67 (61.5%) females and 20 (62,5%) males (p=0.69). And clinical or subclinical symptoms of depression were found in 36 (33.0%) females and 13 (40.6%) males (p=0.73). The mean HADS scores for anxiety were 8.62 [SD 4.61] in males (8.09 [SD 5.0] in 2020; p=0.74) and 9.55 [SD 4.62] in females (9.05 [4.69] in 2020; p=0.66), which corresponded to the level of subclinical severity in the sample. The mean HADS scores for depression were 6.47 [SD 4.10] in males (6.16 [SD 3.21] in 2020; p=0.17) and 5.87 [SD 4.48] in females (6.40 [3.57] in 2020; p=0.021), which corresponded to the normal level in the sample. The frequency of clinically pronounced symptoms of depression (18.4% in 2024 vs. 11.8% in 2020; p=0.118) and anxiety (41.8% in 2024 vs. 32.6% in 2020; p=0.108) did not statistically differ in the samples.

**Conclusions:**

Consistently high rates of anxiety and depression were found among medical students despite the end of the pandemic. This reflects the relevance of further study of the impact of various stressors on the mental well-being of medical students. The results of the study confirm the high vulnerability of medical students to minor mental disorders, which requires the introduction of screening and prevention strategies at the individual and institutional levels.

**Disclosure of Interest:**

None Declared

